# Disease burden of rheumatoid arthritis in the context of population aging: A comparative analysis of China, Japan, the United States and India

**DOI:** 10.1515/rir-2026-0035

**Published:** 2026-10-22

**Authors:** Si Yang, Jiashun Zeng

**Affiliations:** School of Clinical Medicine, Guizhou Medical University, Guiyang, Guizhou Province, China; Department of Rheumatology and Immunology, The Affiliated Hospital of Guizhou Medical University, Guiyang, Guizhou Province, China

**Keywords:** rheumatoid arthritis, disease burden, population aging, cross-national comparison, projection

## Abstract

**Background and Objectives:**

As worldwide population aging accelerates, the burden of rheumatoid arthritis (RA) continues to increase. This study compared the spatiotemporal trends and underlying drivers of the RA burden across four countries with different levels of socioeconomic development under population aging.

**Methods:**

Using data from the Global Burden of Disease (GBD) 2021 study, we analyzed age-standardized rates of incidence, prevalence, mortality, and disability-adjusted life years (DALYs) for RA in China, Japan, the United States, and India from 1990 to 2021. Temporal trends were assessed using Joinpoint regression. A decomposition model was applied to quantify the contributions of population growth, population aging, and epidemiological changes to the overall burden. Spearman correlation analysis was performed to evaluate the association between RA burden indicators and the Sociodemographic Index (SDI). The Nordpred model was employed to project age-standardized prevalence rates among individuals aged 60 years and older up to 2046.

**Results:**

From 1990 to 2021, the numbers of incident cases, prevalent cases, and DALYs attributable to RA increased substantially in China, the United States, and India (*e.g*., prevalent cases in China rose from 2.04 million to 4.76 million, and DALYs increased from 403,000 to 834,000), whereas the corresponding trends in Japan remained relatively stable. Age-standardized DALY rates increased significantly in China and India but exhibited a declining trend in Japan. Decomposition analysis revealed that the rise in RA burden in China was jointly driven by population aging and population growth, while in Japan, population aging alone accounted for nearly the entire increase. RA prevalence showed a strong positive correlation with SDI (*r* = 0.82, *P* < 0.001). Projections indicate that by 2046, the age-standardized prevalence of RA among older adults will continue to rise in China, the United States, and India, whereas Japan is expected to reach a plateau.

**Conclusion:**

The burden of RA remains substantial in China, the United States, and India, and has shown a continuous upward trend, with older women being at particularly high risk. Population aging and population growth are the main driving factors. These findings highlight the need to establish a targeted comprehensive prevention and control system for RA among older adults and formulate differentiated, forward-looking health policies aligned with population aging trends to mitigate the future disease burden.

## Introduction

Rheumatoid arthritis (RA) is a chronic autoimmune disease characterized primarily by persistent synovitis and joint destruction,^[1]^ often leading to chronic pain, functional impairment, and disability. Middle-aged and older patients with RA not only face reduced quality of life and loss of work capacity,^[2]^ but are also at increased risk of mortality and greater loss of healthy life years.^[3,4]^ Although novel drugs such as biologics have improved disease control, their immunosuppressive effects may increase the risk of infections and malignancies, and their high costs also impose a new economic burden on both patients and society.^[5]^

The 21st century has witnessed accelerating population aging. The proportion of persons aged ≥ 65 years is projected to reach 16% by 2050.^[6]^ This trend has contributed to the growing burden of chronic diseases such as RA. Differences in demographic structure, economic development, and healthcare systems across countries profoundly shape the epidemiological patterns and disease burden profiles of RA.

Using data from the Global Burden of Disease (GBD) 2021 study, we included China, Japan, the United States, and India, representing different stages of socioeconomic development. We systematically analyzed the dynamic changes in the incidence, prevalence, mortality, and disability-adjusted life years (DALYs) of RA from 1990 to 2021. Through trend analysis and decomposition analysis, we explored the contributions of the Sociodemographic Index (SDI) and population aging to the RA burden. This study aimed to characterize the heterogeneity of the RA burden across four countries under population aging and to provide evidence for developing targeted prevention and control strategies tailored to different national contexts.

## Methods

### Data Source

Data were obtained from the GBD 2021 study developed by the Institute for Health Metrics and Evaluation (IHME). GBD 2021 constitutes the most comprehensive and detailed assessment of global health to date, providing publicly available estimates for 369 diseases and injuries across 204 countries and regions from 1990 to 2021, including incidence, prevalence, mortality, years lived with disability (YLDs), years of life lost, and DALYs. We extracted RA-related data for four representative countries: China, Japan, the United States, and India.

### Definition of Disease and Evaluation Indicators

In the GBD 2021 study, the RA case definition was based on the International Classification of Diseases (ICD) clinical codes: 714–714.3 and 714.8–714.9 (ICD-9); M05–M06.9 and M08.0–M08.8 (ICD-10).^[7]^ The core disease burden indicators included age-standardized rates of incidence, prevalence, mortality, and DALYs. To account for differences in population age structure, all rates were age-standardized using the GBD 2021 standard population.

Disease burden was analyzed at two levels: (1) an all-age analysis assessing the overall trends in RA burden from 1990 to 2021 across the four countries, and (2) a subgroup analysis focusing on older adults aged ≥ 60 years. Based on the 5-year age-group data from GBD, we aggregated eight age groups, from 60–64 years to ≥ 95 years, to further assess the impact of population aging. Furthermore, we used the Sociodemographic Index (SDI; range 0–1) from GBD 2021 to measure national development levels. The SDI is a composite index combining per capita income, average years of education, and total fertility rate. Countries and regions are categorized by SDI into five levels: low, low-middle, middle, high-middle, and high.

### Statistical Analysis

All data processing, statistical analyses, and figure generation were performed using RStudio (version 4.5.0).

This study employed multiple models to quantify the longterm trends, driving factors, and future projections of RA disease burden. First, the estimated annual percentage change (EAPC) was used to assess the overall trend in age-standardized rates from 1990 to 2021. The EAPC was calculated *via* a log-linear regression model. An increasing trend was considered statistically significant when the lower limit of the 95% CI of the EAPC was > 0. The Joinpoint regression model was applied to automatically identify statistically significant inflection points (joinpoints) in the trends, calculate the annual percent change (APC) for each segment, and derive the average annual percent change (AAPC) for the entire period.

To identify the drivers of changes in disease burden, the demographic decomposition method was employed to attribute the changes in the number of prevalent cases, incident cases, and DALYs from 1990 to 2021 to three aspects: population growth, population aging, and epidemiological change (changes in age-specific disease rates). Furthermore, Spearman’s rank correlation analysis was performed to assess the associations between the SDI and age-standardized prevalence, mortality, and DALY rates among older adults across all GBD countries and territories in 2021. Frontier analysis was applied to visualize the relationship between expected and observed disease burden at different SDI levels.

Based on historical data from 1990 to 2021, We used the Bayesian age-period-cohort (BAPC) model and the Nordpred method to project the age-standardized prevalence of RA among individuals aged 60 years and older in China, Japan, the United States, and India from 2026 to 2046.

## Results

### Trends of RA Disease Burden in China, Japan, the United States and India, 1990–2021

Between 1990 and 2021, the magnitude and temporal trends of the RA burden differed substantially across China, Japan, the United States, and India (Table 1). In terms of prevalence and incidence, in 2021, the number of prevalent RA cases was highest in China (4.76 million), followed by India (2.77 million), the United States (1.46 million), and Japan (696,000). The ranking of incident cases in the same year was similar. The EAPC from 1990 to 2021 indicated increasing trends in age-standardized rates of both prevalence and incidence in India (EAPC for prevalence: 1.315, for incidence: 1.167), the United States (0.768, 0.734), and China (0.588, 0.612), while Japan experienced a slight decline (-0.178, -0.248). In contrast, age-standardized RA mortality rates declined in all four countries. Japan demonstrated the most substantial decrease (EAPC = -2.739), followed by the United States (-1.191), China (-0.384), and India (-0.242).

**Table 1:** Absolute numbers and estimated annual percentage changes (EAPCs) of rheumatoid arthritis burden indicators in China, the United States, India, and Japan, 1990–2021

Indicator	Measure	China	Japan	United States of America	India
Prevalence	Number (1990)	2,041,681.8	525,814.4	751,904.12	818,572.72
		(1,746,912.69, 2,391,022.36)	(458,565.13, 609,512.61)	(689,983.66, 817,799.66)	(697,518.25, 969,037.2)
	Number (2021)	4,755,486.53	695,657.98	1,459,692.28	2,766,748.56
		(4,141,219.49, 5,452,492.35)	(618,010.82, 790,455.08)	(1,346,054.78, 1,594,920.61)	(2,412,595.78, 3,215,435.13)
	EAPC (1990-2021)	0.588	-0.179	0.768	1.315
		(0.553, 0.624)	(-0.286,-0.071)	(0.679, 0.857)	(1.205, 1.426)
Incidence	Number (1990)	127,825.77	31,521.16	43,581.63	63,046.74
		(111,476.76, 145,914.45)	(27,205.79, 36,783.15)	(39,473.12, 48,167.08)	(54,893.01, 72,108.01)
	Number (2021)	247,307	32,060.11	81,504.81	198,510.96
		(216,204.83, 282,997.58)	(27,599.35, 37,007.64)	(73,804.62, 90,798.19)	(174,796.13, 226,623.7)
	EAPC (1990-2021)	0.612	-0.248	0.734	1.167
		(0.585, 0.639)	(-0.352,-0.144)	(0.661, 0.807)	(1.082, 1.253)
Deaths	Number (1990)	4776.39	1549.81	1703.22	2909.35
		(3902.58, 5934.4)	(1423.72, 1622.76)	(1558.07, 1781.52)	(2004.69, 4204.93)
	Number (2021)	10,279.11	2211.71	2260.14	7678.59
		(7411.45, 12,614.82)	(1798.88, 2470.25)	(1960.58, 2480.21)	(5624.93, 11,355.89)
	EAPC (1990-2021)	-0.384	-2.74	-1.191	-0.242
		(-0.751,-0.015)	(-3.259,-2.218)	(-1.714,-0.665)	(-0.4,-0.083)
DALYs	Number (1990)	403,058.41	100,774.53	131,750.61	169,250.47
		(307,641.17, 526,243.7)	(79,027.27, 129,604.72)	(100,651.82, 167,292.7)	(128,066.69, 218,649.07)
	Number (2021)	833,817.85	122,653.9	226,825.77	503,360.48
		(621,520.18, 1,083,523.47)	(93,498.61, 158,301.15)	(170,238.06, 291,787.86)	(382,564.35, 638,990.18)
	EAPC (1990-2021)	0.169	-0.849	0.281	0.602
		(0.052, 0.286)	(-0.911,-0.788)	(0.143, 0.419)	(0.516, 0.689)
YLDs	Number (1990)	275,235.12	69,396.17	97,779.69	107,267.28
		(184,228.65, 394,823.72)	(47,729.37, 98,203.37)	(67,478.51, 132,703.8)	(71,169.7, 153,875.08)
	Number (2021)	631,813.82	90,606.3	185,860.46	357,498.09
		(429,315.83, 883,990.62)	(62,769.59, 125,773.2)	(129,757.43, 251,039.66)	(243,088.21, 496,359.5)
	EAPC (1990-2021)	0.596	-0.155	0.735	1.319
		(0.559, 0.632)	(-0.26,-0.051)	(0.642, 0.828)	(1.21, 1.429)

EAPCs, estimated annual percentage changes; YLDs, years lived with disability; DALYs, disability-adjusted life years.

Regarding disability burden, the number of DALYs in 2021 ranked from high to low as China (834,000), India (503,000), the United States (227,000), and Japan (123,000). The EAPC for age-standardized DALY rates showed an upward trend in India (0.602), the United States (0.281), and China (0.169), whereas Japan showed a downward trend (EAPC = −0.849), although its absolute number of DALYs continued to increase over the period. Trends in YLDs were similar to those observed for DALYs.

Overall, India exhibited the most rapid growth in most indicators. The burden continued to increase in China and the United States but at a relatively moderate pace. Japan was the only country in which all age-standardized indicators remained stable or declined during the study period.

### Age- and Sex-specific Patterns of RA Burden in the Four Countries, 2021

In 2021, China, Japan, the United States, and India exhibited similar age- and sex-specific patterns of RA burden, although marked differences existed in absolute levels (Figure 1). Regarding incidence and prevalence, both rates in all four countries increased with age, peaking in the 60–74-year age group before declining. The numbers of incident and prevalent cases were highest in China and India during the peak age groups (*e.g*., prevalent cases in China among those aged 60–69 years exceeded 600,000), followed by the United States, with Japan having the lowest figures. The incidence and prevalence rates for females were consistently higher than those for males across all age groups, with the female-to-male ratio reaching approximately 1.5 to 2 times during the peak ages.

**Figure 1:**
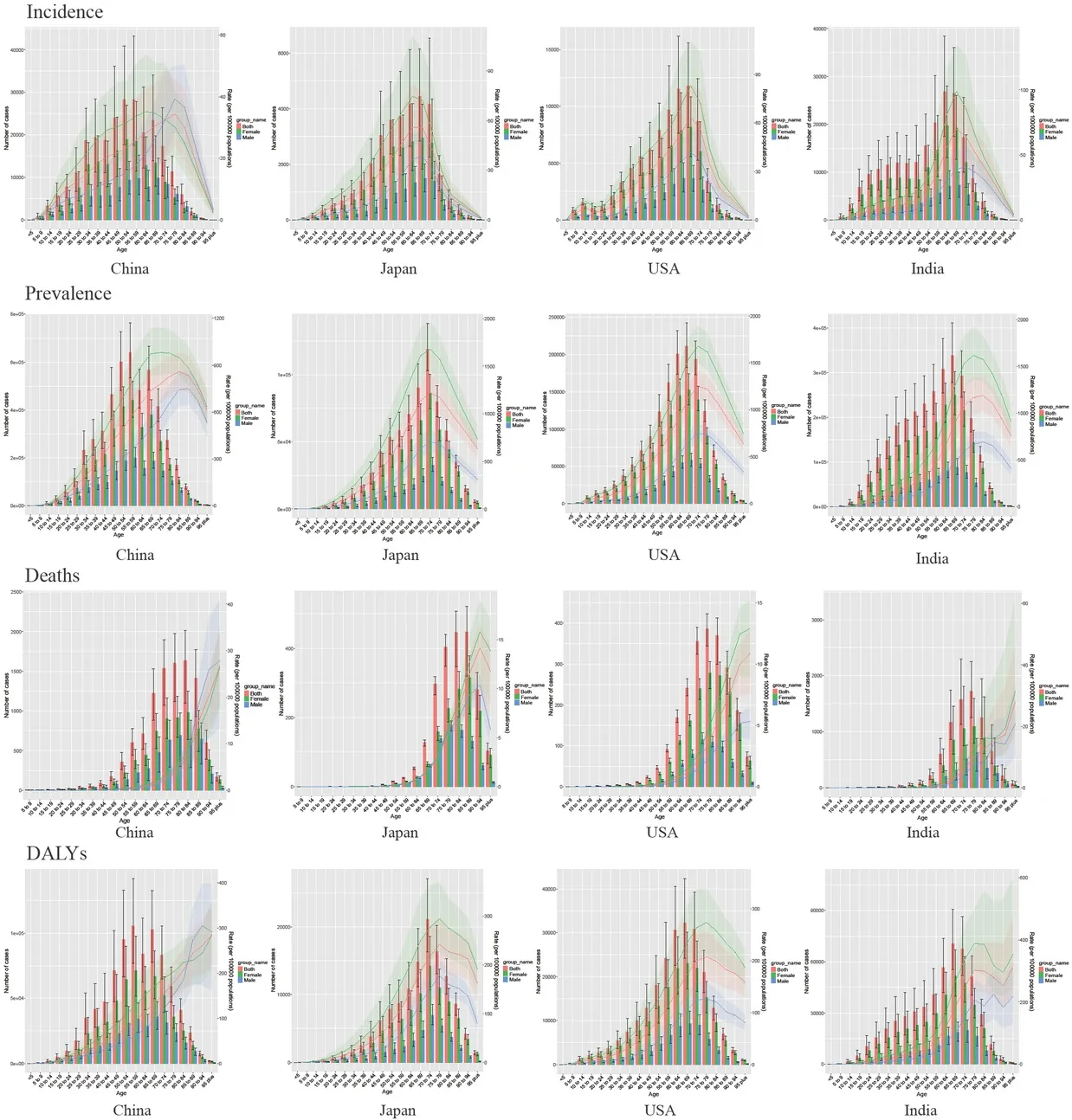
Age- and sex-specific incidence, prevalence, mortality, and DALY rate of rheumatoid arthritis in China, Japan, the United States and India, 2021. DALY: disability-adjusted life year.

In terms of mortality and overall burden, the mortality peak occurred later, concentrated primarily in the older age group of 70–84 years. The number of deaths was highest in China and India (*e.g*., > 1500 deaths among those aged 75–79 years in China), while figures for the United States and Japan were notably lower. Sex differences in mortality were relatively modest, with a female-to-male ratio of approximately 1.2 to 1.5 times. DALYs peaked among individuals aged 60–79 years. The DALYs in China far exceeded those of the other three countries (*e.g*., > 100,000 DALYs among those aged 65–69 years). Similarly, DALY burden was consistently higher among females, with peak values reaching approximately 1.5–2 times those observed in males.

### Joinpoint Regression Analysis of Temporal Trends in Age-standardized RA Disease Burden Across the Four Countries, 1990–2021

From 1990 to 2021, the age-standardized disease burden of RA in the four countries exhibited distinct temporal trends (Figure 2). Both age-standardized incidence and prevalence rates demonstrated an overall upward trend. The age-standardized incidence rate (ASIR) increased steadily in India (AAPC = 2.09%), China (1.53%), and the United States (1.16%). In particular, India experienced an accelerated increase after 2011 (APC = 2.95%), while Japan remained stable (AAPC near zero).

**Figure 2:**
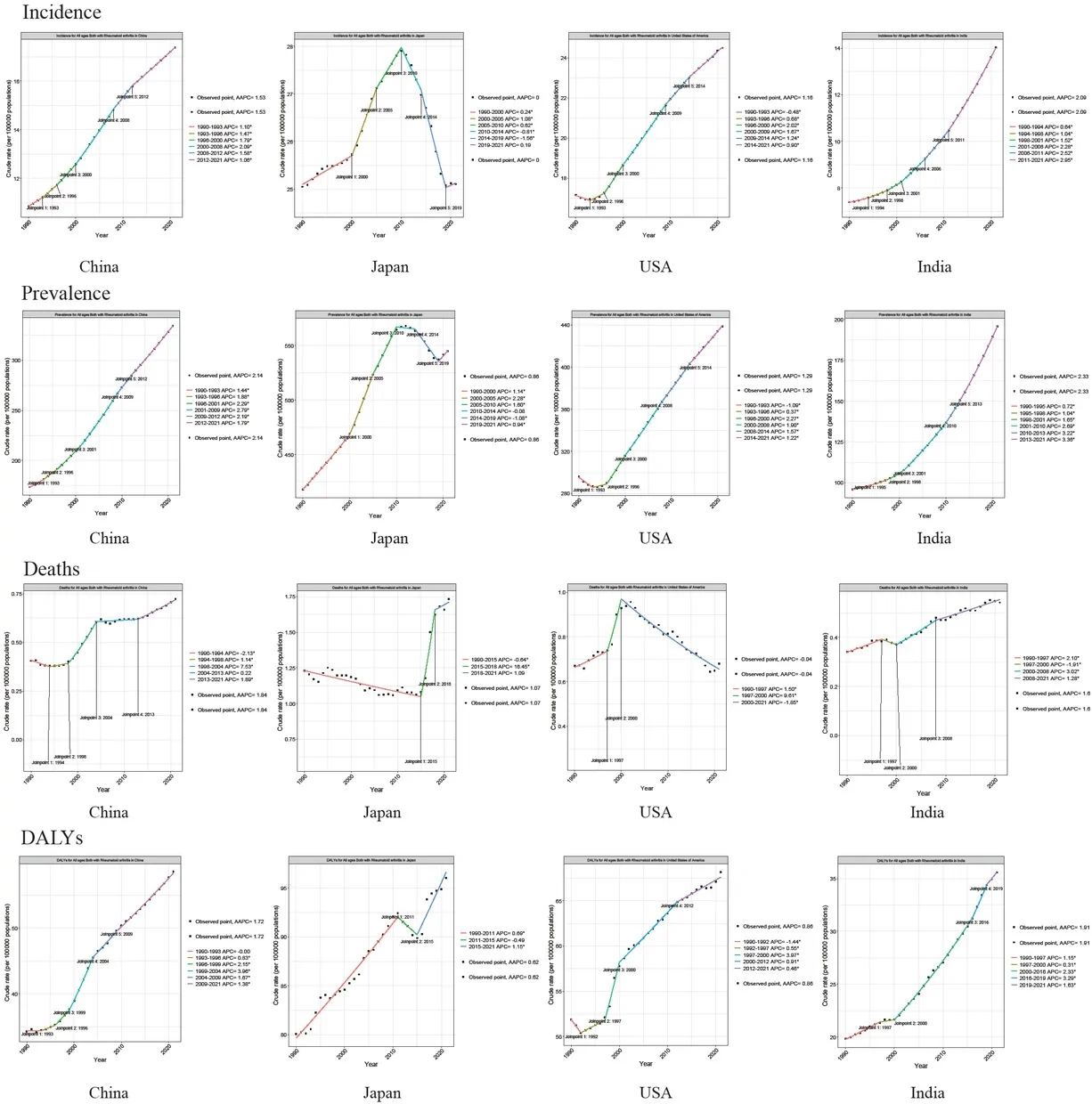
Time-trends in age-standardized incidence, prevalence, mortality, and DALY rates for rheumatoid arthritis in China, Japan, the United States and India, 1990–2021. DALY: disability-adjusted life year.

The increase in the age-standardized prevalence rate (ASPR) was more pronounced. India (AAPC = 2.33%) and China (2.14%) exhibited the fastest growth, with India showing a further acceleration after 2013 (APC = 3.36%). The United States (1.29%) and Japan (0.86%) showed more gradual increases, with Japan showing a transient decline during 2014–2019 (APC = -1.08%).

Divergent trends were observed for mortality and composite burden. The age-standardized mortality rate (ASMR) showed an overall increasing trend in India (AAPC = 1.60%) and China (1.84%), albeit with significant fluctuations. In contrast, the United States showed a continuous decline since 2000 (APC =-1.85%), indicating sustained reductions in RA mortality. Japan, after a long-term decline, showed a transient rebound during 2015–2018 (APC = 16.45%). The age-standardized DALY rate exhibited distinct stage-specific trends across the four countries. India (AAPC = 1.91%) and China (1.72%) showed rapid growth, while the United States (0.86%) and Japan (0.62%) experienced relatively moderate increases. Of note, Japan demonstrated a nonlinear pattern: an initial increase, followed by a decline, and then a subsequent rise. The increase in DALY rates in China and India exceeded that in their mortality rates, suggesting that nonfatal disability contributed increasingly to the overall disease burden.

### Decomposition Analysis of Changes in RA Disease Burden in the Four Countries, 1990–2021

Decomposition analysis indicated that between 1990 and 2021, population aging and population growth were the primary drivers of increases in the numbers of prevalent cases, incident cases, and DALYs across the four countries.

Although the contribution of epidemiological changes was relatively small, the dominant drivers varied substantially across countries (Figure 3). At the overall level, Japan exhibited a typical aging-dominated pattern across all indicators, with nearly all increases in disease burden attributable to population aging. China displayed a dual pattern driven by both population aging and population growth, in which aging made a particularly prominent contribution to the increase in prevalent cases, whereas the growth in incident cases and DALYs was jointly driven by both factors, indicating a transition toward an aging-dominated pattern. India displayed a population growth-dominated pattern, where population growth was the primary driver, with aging playing a secondary role. The United States occupied an intermediate position, with relatively balanced contributions from population aging and population growth, and could be described as a balanced-driving pattern.

**Figure 3:**
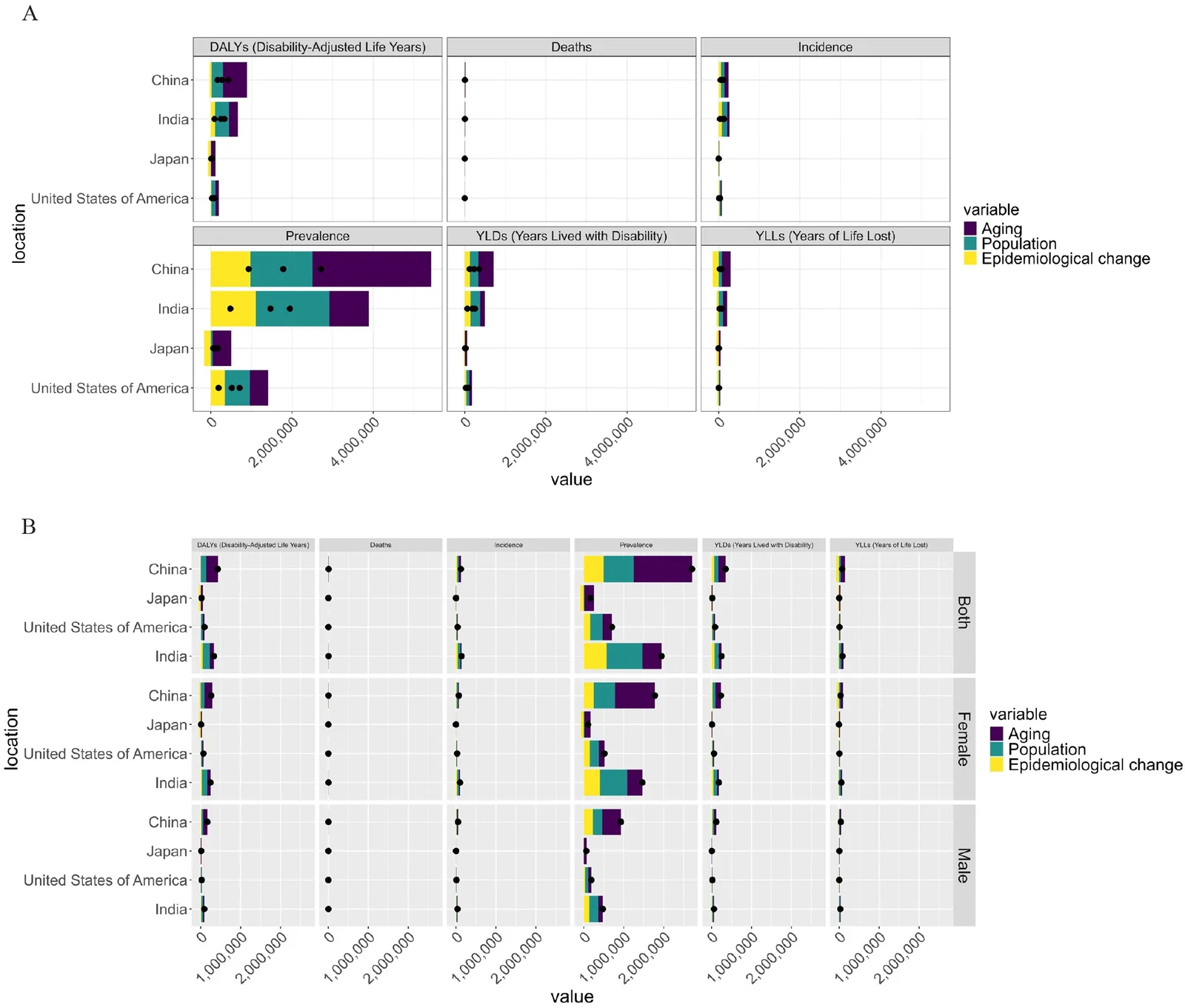
Decomposition analysis of changes in rheumatoid arthritis disease burden in China, Japan, the United States, and India, 1990–2021. (A) overall decomposition; (B) sex-stratified decomposition

Sex-stratified results aligned with the overall trends. The factors driving the increase in disease burden were generally similar between females and males but exhibited more pronounced differences in some countries. For instance, a higher proportion of the increase in prevalent cases and DALYs among Chinese females was attributable to population aging than among males. The increase in disease burden among females in India remained primarily driven by population growth. Japan and the United States maintained consistent sex-specific patterns, characterized by aging-dominated and balanced driving forces, respectively.

### Cross-country Correlations between RA Disease Burden Among Older Adults and the Sociodemographic Index (SDI) in 2021

Cross-country correlation analysis for 2021 revealed a significant positive correlation between the age-standardized prevalence rate (ASPR) of RA among older adults and the SDI (Spearman *ρ*= 0.53, *P* < 0.001) (Figure 4), indicating that countries with higher SDI generally have higher ASPR of RA among older adults. China (high-middle SDI) and India (middle SDI) had age-standardized prevalence rates notably higher than those of other countries within the same SDI category. The United States and Japan (both high SDI) also maintained relatively high prevalence levels worldwide.

**Figure 4:**
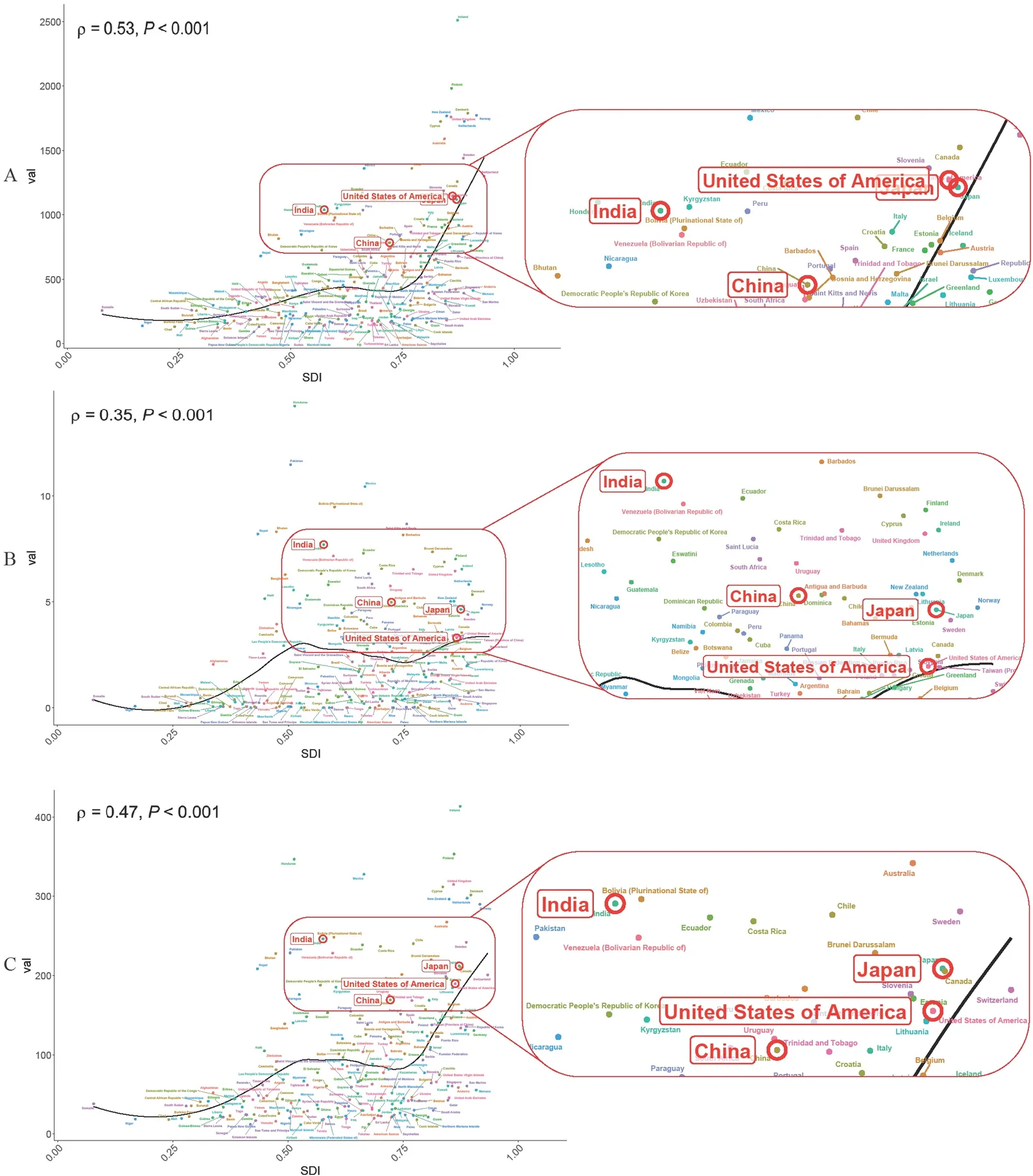
Correlation between age-standardized prevalence, deaths, and DALY rates of rheumatoid arthritis in older adults and the sociodemographic index across countries globally, 2021. DALY: disability-adjusted life year.

In contrast, the age-standardized death rate (ASDR) of RA among older adults showed a weaker correlation with SDI (*ρ* = 0.35, *P* < 0.001). China and India exhibited higher ASDRs than the average within their respective SDI categories, whereas the United States and Japan were close to the average level among high-SDI countries, suggesting a relative advantage in controlling RA mortality. The age-standardized DALY rate of RA among older adults showed a moderate positive correlation with SDI (*ρ* = 0.47, *P* < 0.001). Both China and India had higher DALY rates within their respective SDI categories, and the United States and Japan also maintained relatively high levels in the high SDI group. These findings suggest that, regardless of the stage of social development, the four countries studied all face a substantial health loss attributable to RA.

### Frontier Analysis of Age-standardized Prevalence and DALY Rates for RA Among Older Adults, 2021

Frontier analysis showed that the ASPR of RA among older adults generally increased across countries with higher SDI levels (Figure 5A). Prevalence was generally low in low-SDI countries and increased markedly in middle- and high-SDI countries. China (high-middle SDI) and India (middle SDI) had prevalence rates higher than those of other countries within their respective SDI categories. Among high-SDI countries, considerable heterogeneity was observed: for instance, the United States remained on an upward trajectory, while Japan and some European countries had already shown a declining trend.

**Figure 5:**
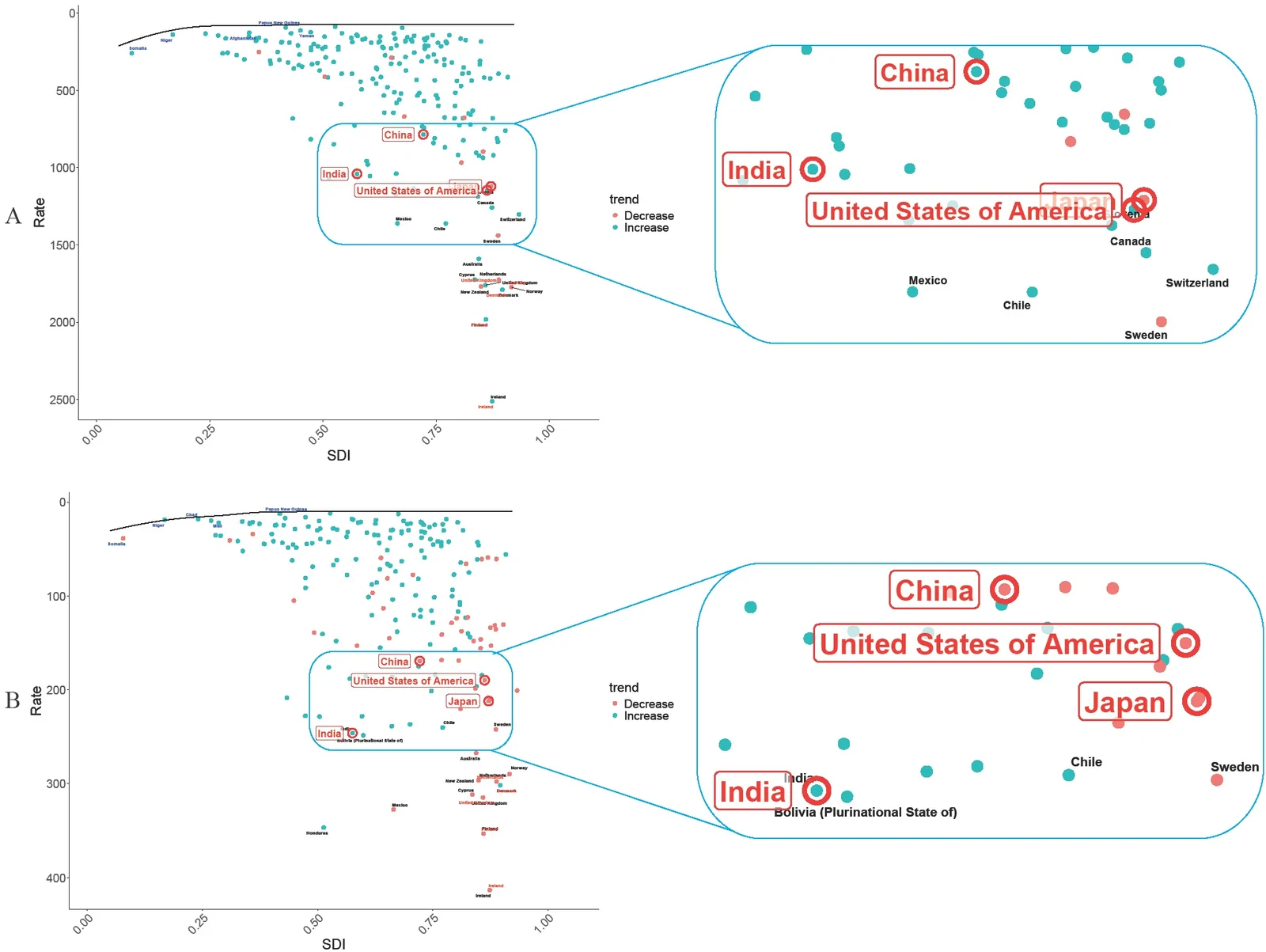
Frontier analysis of global age-standardized prevalence (A) and age-standardized DALY rate (B) of rheumatoid arthritis in older adults and sociodemographic index in 2021. DALY: disability-adjusted life year.

The distribution of age-standardized DALY rates of RA among older adults was similar to that of prevalence, but the patterns of change were distinct (Figure 5B). The burden was relatively low in low-SDI countries. In middle-SDI countries (*e.g*., India), the burden was associated with progressively higher DALY rates across increasing SDI levels. In high-middle and high-SDI countries, most countries exhibited lower DALY rates. Although the burden levels in China and the United States were moderate, they had already shown a declining trend. Japan, within the high-SDI range, exhibited a relatively low burden level that continued to decrease. In contrast, India stood out by maintaining a clear upward trend, with a DALY rate exceeding that of most other middle-SDI countries.

### Projected Age-standardized Prevalence of RA Among Adults Aged ≥ 65 Years in the Four Countries, 2026–2046

Projection results indicate that between 2026 and 2046, the ASPR of RA among older adults will continue to rise in China, the United States, and India, with larger increases among females than males (Figure 6). Both China and the United States are projected to experience stable, gradual growth, with case numbers accumulating in parallel, reflecting an expanding pool of prevalent RA cases driven by population aging. India is expected to experience the steepest increase; female ASPR is projected to increase more rapidly than that of any other country, and absolute case numbers will grow most rapidly, indicating a combination of a high baseline burden and rapid future growth. In contrast, ASPR among Japanese males will remain virtually flat, while that among females is projected to decline modestly after approximately 2030.

**Figure 6:**
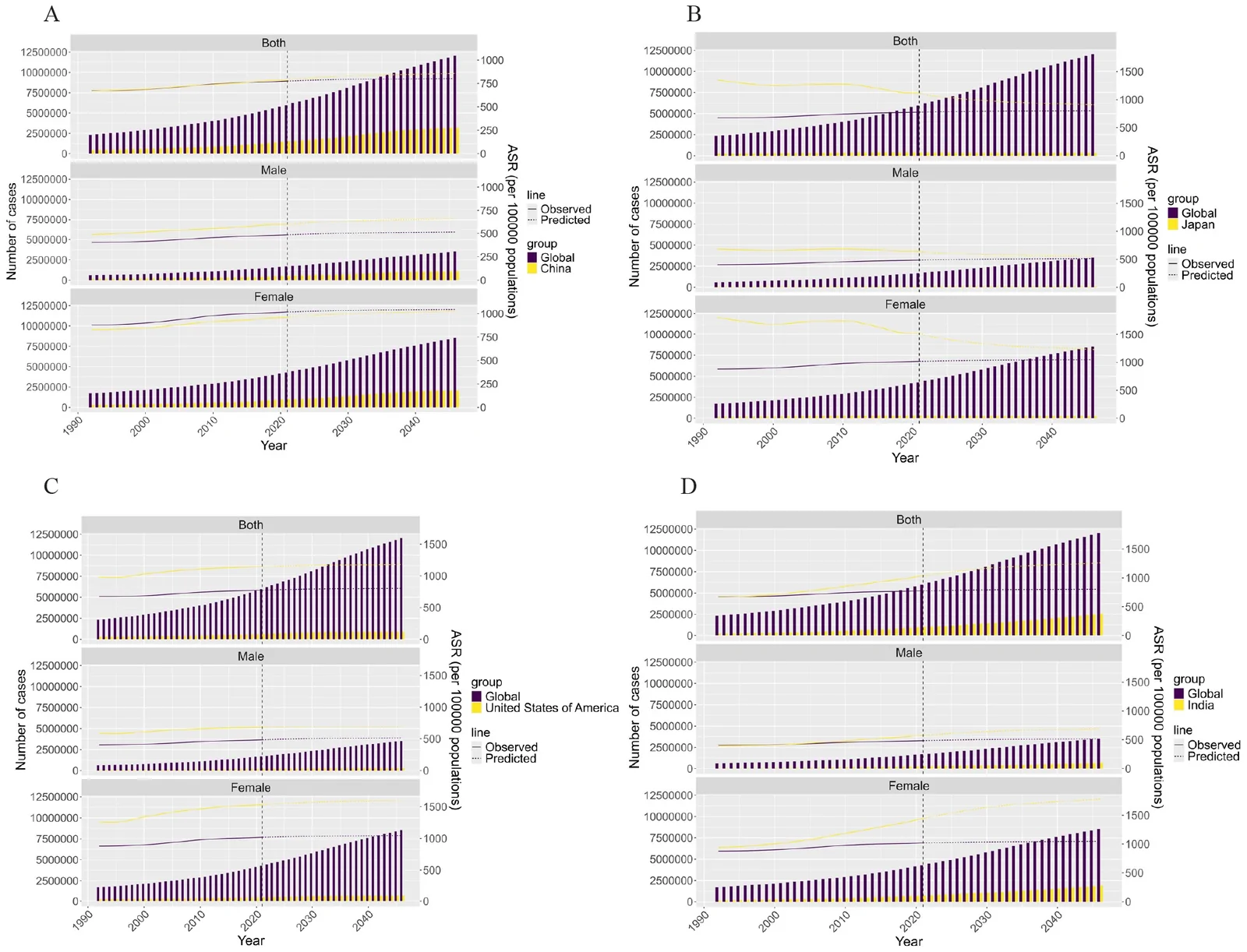
Projected trends in age-standardized prevalence and number of prevalent cases of rheumatoid arthritis among adults aged ≥ 65 years in China (A), Japan (B), the United States (C), and India (D), 2026–2046.

Although case numbers in Japan will still grow, the pace will be markedly slower than in the other three countries, reflecting slow case accumulation despite persistently high prevalence levels, which suggests that the RA burden in Japan may be entering a relatively stable phase.

Overall, China, the United States, and India are projected to face continued concurrent increases in both prevalence rates and absolute case numbers, whereas Japan, although experiencing continued case growth, will see its ASPR stabilize or decline slightly with a substantially slower rate of increase than the other three countries. This suggests that Japan is gradually transitioning into a phase of stable but persistently high disease burden. The divergent future trends in RA burden among these four countries indicate that developing economies will continue to face the challenge of rapid growth, whereas high-SDI countries are entering a relatively stable plateau phase.

## Discussion

This study systematically assessed temporal trends in the burden of RA in China, Japan, the United States, and India from 1990 to 2021. Against the backdrop of an overall decline in the ASMR and age-standardized DALY rate for RA observed in the GBD study, significant heterogeneity was observed among these countries: the incidence rate and disease burden in China and India continued to increase, whereas changes in the United States were comparatively modest. Japan exhibited a pattern of increasing total burden but decreasing age-standardized risk, whereby the absolute number of DALYs increased, whereas the age-standardized DALY rate declined, indicating that population aging and changes in disease risk simultaneously shaped the evolution of its disease burden. These findings are consistent with previous GBD studies^[8,9]^ and indicate that differences in RA burden are influenced by multiple factors, including population aging, disease management systems, and healthcare accessibility. Moreover, the age and sex patterns of RA were generally similar across the four countries: the burden was consistently higher in females than in males, and both the incidence rate and the DALY burden peaked in the 60–79 age group, reflecting female susceptibility and the concentration of RA in older adults.^[10,11]^ These findings highlight the importance of prioritizing middle-aged and older women in RA prevention and disease management strategies.

Joinpoint regression analysis revealed that China and India experienced the fastest increases in RA incidence rates, which may be attributable to the combined effects of population aging and improved access to diagnosis and treatment. A transient decline in incidence was observed in the United States during the early 1990s, potentially associated with the implementation of the 1987 revised classification criteria.^[12]^

However, the burden of RA remained at a relatively high level due to lifestyle factors such as obesity and smoking.^[13,14]^ Notably, the EAPC and Joinpoint analysis yielded opposite directional findings for Japan’s age-standardized DALY rate: the EAPC indicated an overall decline, while Joinpoint showed a slight increase. This discrepancy arises because the EAPC reflects the overall trend across the entire study period, whereas Joinpoint analysis is capable of identifying stage-specific changes. The age-standardized DALY rate in Japan rebounded during the later part of the study, resulting in a positive AAPC. Thus, the RA burden in Japan followed a pattern of decreasing age-standardized risk but increasing total burden, reflecting the combined effects of population aging and changes in disease risk. Previous studies have suggested that Japan’s long-term implementation of standardized RA management strategies may be associated with improved disease control.^[15, 16, 17, 18, 19]^ However, whether these strategies have directly contributed to changes in the national disease burden remains to be established. Although the age-standardized mortality rates in the four countries generally decreased—a trend possibly related to updates to diagnostic and treatment guidelines,^[20,21]^ the application of biologics and targeted therapies, and improvements in comprehensive management—age-standardized DALY rates continued to rise in China and India. These findings suggest that, despite reductions in mortality, further efforts are needed to reduce functional impairment and improve quality of life among patients with RA.

Decomposition analysis showed that increases in RA disease burden were primarily driven by population aging and population growth, whereas changes in disease epidemiology contributed relatively little. This suggests that changes in age-specific disease risk were relatively modest, and the increase in disease burden largely reflects the expansion and aging of the susceptible population rather than substantial changes in the underlying disease risk. Country-specific analysis showed that the driving patterns differed across the four countries: the growth in prevalent cases in China was mainly driven by population aging, while the increase in DALYs was driven by both population aging and population growth. In Japan, the increase in all indicators was almost entirely attributable to aging, consistent with its status as a “super-aged” society.^[22]^ The rising burden in India was primarily driven by population expansion, suggesting that population growth was the predominant contributor to the increasing burden. In contrast, the United States exhibited a balanced influence of both aging and population growth. Furthermore, the increase in disease burden among women was more significantly affected by aging, which may be related to longer life expectancy, declining estrogen levels, and differences in immune responses.^[23, 24, 25]^ Therefore, in a context where population aging is the dominant driver of disease burden growth, relying solely on treatment efforts is unlikely to reverse the overall trend. In highly aged societies, prevention and control strategies should prioritize the optimization of integrated care and functional maintenance for older adults with RA.^[26,27]^ Public health strategies should shift toward an integrated prevention–management–rehabilitation continuum, with an emphasis on early screening, integrated chronic disease management for older adults, and functional maintenance to address these structural challenges.

Differences in population structure, economic development, and healthcare systems across countries lead to distinct RA prevention and control approaches. China is undergoing rapid population aging, with the proportion of people aged 65 and above expected to approach 30% by the mid-century,^[28,29]^ resulting in dual pressures from accelerated aging and uneven distribution of medical resources. Japan, one of the earliest countries to enter a “super-aged society, ” already has 29% of its population aged 65 or older.^[30,31]^ The high burden of comorbidities among older adults with RA continues to strain the Japanese healthcare system.^[32]^ In contrast, the United States, a multicultural, high-income country with substantial immigration,^[33]^ possesses a relatively advanced healthcare system, yet still struggles with disparities in resource allocation and high out-of-pocket costs.^[34,35]^ India is projected to have 300 million people aged 60 and above by 2051.^[36]^ Given its rapidly aging population, limited healthcare resources, and large population base,^[37]^ India may face a rapid rise in the RA burden in the future. Our projections suggest that, over the next two decades, the burden of RA among older adults will continue to increase in China, the United States, and India, while remaining stable in Japan. Notably, if effective interventions are lacking, India may experience particularly rapid growth in RA cases, placing considerable pressure on its healthcare system. For China, although healthcare reforms have improved treatment accessibility, the sheer size and continued growth of the elderly population will drive an increase in both the total number of patients and the disability burden, a trend that will be difficult to reverse in the short term.

This study found that countries with a higher SDI did not necessarily have a lower RA disease burden. For instance, although countries such as the United States and Japan have relatively abundant medical resources, their RA burden remains high. Apart from the accumulation of prevalent cases and growing disability burden due to prolonged survival, this phenomenon may also be influenced by factors such as changes in diagnostic criteria, improved disease recognition, and differences in ethnic and genetic backgrounds. For example, the 2010 ACR/EULAR classification criteria have higher sensitivity for early disease recognition compared to the 1987 criteria, which may increase the detection rate of RA cases; some ethnic minority populations in the United States have also been reported to have a higher risk of RA.^[38]^ Therefore, the higher RA burden in high-SDI countries may not fully reflect a true increase in disease risk, but rather may be attributable to a combination of factors. Given the lack of data on diagnostic criteria usage, ethnicity-stratified information, and individual medical records in the GBD database, the present study could not directly test these hypotheses. On the other hand, even in high-SDI countries, issues such as disparities in healthcare coverage, high out-of-pocket expenses,^[39]^ and insufficient access to sustained, evidence-based treatment may still affect disease control. Comorbidities such as obesity and cardiovascular disease may also exacerbate functional impairment.^[40,41]^ Therefore, relying solely on expanding diagnosis and treatment coverage is unlikely to substantially reduce the overall burden of RA. Future efforts should focus on optimizing functional recovery, comorbidity management, and long-term health maintenance.

This study indicates that population aging is the principal structural factor driving the increasing burden of RA, while its impact is influenced by the level of socioeconomic development and health system capacity. In China and Japan, population aging was the main contributing factor to the increase in the number of RA patients and the absolute DALY burden, consistent with the trend of aging driving the rising chronic disease burden.^[42,43]^ Notably, among older adults with RA, the risk of comorbidities increases with age, further complicating disease management. Based on the four-country comparison, this study categorizes RA prevention and control models into four types. First, the resource-intensive and well-covered model (the United States): characterized by a high-level healthcare system that effectively controls mortality and disability rates, yet issues of inequitable access and high cost burden persist. Second, the aging-adapted, function-maintenance model (Japan): in an ultra-aging society, this model exhibits a pattern of increasing patient numbers but decreasing age-standardized disease burden, with a focus on elderly patients with multimorbidity,^[44,45]^ emphasizing integrated care and functional maintenance across the life course. Third, the rapid-growth, resource-limited model (India): under the dual constraints of rapid population growth and insufficient healthcare resources, patient growth outpaces health system capacity. Fourth, the dual-pressure, transition-optimization model (China): under the pressure of accelerated aging and a large population base, DALYs continue to rise despite improved treatment accessibility, indicating a need to strengthen disability prevention and functional maintenance within disease management. Correspondingly, prevention and control strategies should be differentiated by type: resource-intensive countries should optimize equitable resource allocation and comorbidity management; super-aged societies should focus on functional maintenance and integrated care,^[46,47]^ promoting integrated chronic disease management systems; resource-limited countries should prioritize early screening^[48]^ and access to basic diagnosis and treatment; and transitional countries can draw on the experiences of other nations, advance pharmaceutical policy and healthcare financing reforms, and build comprehensive prevention and control systems suited to their national contexts. In summary, in the context of ongoing population aging, RA prevention and control should be expanded beyond optimized pharmacotherapy to encompass life-course management. Through early identification, comorbidity management, and functional rehabilitation, a sustainable framework for healthy aging can be established to achieve the dual goals of extending survival and improving quality of life.

This study integrated data from the GBD 2021 study with multiple analytical approaches, providing a quantitative basis for cross-national comparisons and informing public health policy. However, several limitations should be acknowledged. First, GBD disease burden estimates rely on surveillance data and statistical models, which may introduce uncertainty in regions with insufficient data. Among older adults, a high burden of comorbidities may affect the diagnosis and attribution of RA. Updates to RA classification criteria during the study period may also have influenced long-term trend analyses. In addition, this study did not incorporate health system factors such as medication accessibility and healthcare utilization. Therefore, the findings should be interpreted primarily as reflecting trends in disease burden and relative differences between countries, and future validation using real-world data is warranted.

From 1990 to 2021, substantial differences in RA burden were observed across the four countries included in this study. The continuous increase in the number of patients and disease burden in China, the United States, and India suggests that, against the backdrop of accelerated population aging, their disease prevention strategies and health resource allocation models may require further optimization. Japan, on the other hand, exhibited a characteristic of “increasing total burden but decreasing age-standardized risk,” indicating that a well-established disease management and healthcare delivery system may contribute to more favorable disease outcomes at the individual level, offering a reference for RA prevention and control in an aging society. Looking ahead, demographic shifts and population ageing are projected to further increase the RA burden in developing economies and rapidly aging societies. Reducing the overall burden of RA not only requires increasing investment in medical resources, but also necessitates optimizing the allocation of health resources and prevention and control pathways. This requires strengthening early detection, guideline-based treatment, and primary rehabilitation capacity; promoting payment reforms and improved access to effective therapies to enhance treatment adherence and long-term health outcomes. Future RA prevention and control efforts should move beyond a treatment-centered approach toward comprehensive health management, with greater emphasis on preserving physical function, managing comorbidities, and promoting healthy aging throughout the life course.
